# Prevalence of congenital heart defect in Guangdong province, 2008-2012

**DOI:** 10.1186/1471-2458-14-152

**Published:** 2014-02-11

**Authors:** Li Wu, Bing Li, Jianhong Xia, Cunwei Ji, Zhijiang Liang, Yuanzhu Ma, Shiyun Li, Yuntao Wu, Youjie Wang, Qingguo Zhao

**Affiliations:** 1 Guangdong Women and Children Hospital, Guangzhou, China; 2 Department of Maternal and Child health, School of Public Health, Tongji Medical College, Huazhong University of Science and Technology, Wuhan, China

**Keywords:** Birth defects, Congenital heart defect, Prevalence

## Abstract

**Background:**

Congenital heart defect (CHD) is the most common major malformations in infants. Little is known about the main epidemiologic characteristics of CHD prevalence in Guangdong province, China. Our study was undertaken to investigate the time trends in the prevalence of CHD in Guangdong province from 2008 to 2012.

**Methods:**

Data were retrieved from the Guangdong Hospital-Based Birth Defects Monitoring System during 2008–2012. All infants more than 28 weeks of gestation and infants up to 7 days of age in monitoring hospitals were monitored. We used prevalence rate to describe the difference in prevalence of CHD between rural and urban areas. Odds ratio (OR) and 95% confidence interval (CI) for CHD were calculated for the rural and urban areas. The CHD rate was calculated on the basis of birth defects per 10,000 births.

**Results:**

A total of 1005052 births were reported to the Birth Defects Monitoring Network of Guangdong Province, of which 5268 cases were diagnosed as CHD. The overall prevalence of CHD was 52.41 per 10 000 births (95% CI: 51.00 ~ 53.83) in provincial-wide, 66.08 per 10 000 births (95% CI: 63.77 ~ 68.39) in urban areas, and 40.23 per 10 000 births (95% CI: 38.52 ~ 41.93) in rural areas. The prevalence of CHD increased with maternal age both in urban areas (*P* < 0.01) and in rural areas (*P* < 0.01).

**Conclusion:**

The increasing trends of CHD prevalence suggest that maternal age and the improvement of diagnosis ability might play a critical role.

## Background

Birth defects are the main causes of early miscarriage, perinatal death, infant death and child disabilities, which have been a global public health issue [1]. Congenital heart defects (CHD) is the most common type of birth defects [2,3], with a wide range of reported birth prevalence estimates. It is the leading cause of infant mortality and morbidity [4-6] associated with birth defects and can result in chronic disability and increased health care costs [7,8].

Epidemiological studies in developed countries have indicated that a prevalence of CHD ranged from 4–10 per 1000 live births [3,9,10]. The prevalence of CHD among fetuses is estimated to be even higher, at 14.6 per 1000 fetuses [11]. Complex CHD is common among fetuses [12] and may result in spontaneous abortion or stillbirth, the total prevalence of CHD among stillbirths and live births may be higher than that reported among live births alone.

The prevalence of CHD has been studied in developed countries for many years [3,4], and it is also studied in the mainland of China [13,14]. However, little is known about the main epidemiologic characteristics of CHD prevalence in Guangdong province. This study was done using the Hospital-Based Birth Defect Surveillance System of Guangdong Province, China. We analyzed the prevalence and characteristics of CHD in perinatal infants in Guangdong province during 2008–2012, in order to provide a scientific basis for developing control measures over prevention of CHD on perinatal infants (from 28 weeks of gestation to a period of 7 days after delivery).

## Methods

Data were obtained from the Birth Defects Monitoring Network of Guangdong Province from 2008 to 2012. The monitoring system is hospital-based registry covering 58 hospitals across the province, and the hospitals at the county level or above were selected to participate. The program primarily monitors infants with birth defects who are born in hospitals. The subjects monitored by the network included live births and stillbirths who were delivered in hospital after at least 28 weeks of gestation. The clinical diagnosis of CHD was diagnosed within 7 days after delivery. Within this period, all diagnosed birth defects were required to be reported. The CHD was identified according to the International Classification of Diseases Clinical Modification Codes, tenth edition (i.e., ICD-10).

CHD was defined as “a gross structural abnormality of the heart or intrathoracic great vessels that is actually or potentially of functional importance [13]. An isolated patent foramen ovale and patent ductus arteriosus were excluded because they are normal findings in perinatal infants.

### Data collection and quality management

A registration form for birth defect cases was used to collect information for CHD cases. In the monitoring hospitals, the health care professionals examined every infant immediately after birth to screen for CHD. The diagnosis and verification must be completed within 7 days after delivery. For those affected by CHD, an inperson interview with the mother and medical record reviews were conducted to gather information by a trained doctor. The information is collected using a standard birth defects registration form. The data about deliveries, the number of perinatal infants, and sex of infants are collected using the Seasonal Report Form. The county-level staff submits the forms using the web-based data reporting system. The forms are checked by senior who are responsible for data quality in the monitoring hospital. When errors were identified, the form should be returned for verification. The monitors at the municipal and provincial levels should check the forms. Finally, the data was sent to the National Center for Birth Defects Monitoring (national center level), where the experts were responsible for the final confirmation of diagnosis, data checking and evaluating, and disease coding, etc. Because our data was obtained from the database of the Birth Defects Monitoring Network of Guangdong Province, and the data was de-identified and analyzed anonymously, our ethics committee waived the need for informed consent. We received permission from the Birth defects Intervention Group of Guangdong Province to use the data.

### Statistical analysis

Residential areas were categorized into rural and urban areas according to mother’s residence address during the pregnancy. The number of deliveries in the hospital after at least 28 weeks’ gestation was used as the denominator, and the number of infants diagnosed with CHD within 7 days of birth was used as the numerator. Prevalence estimates are reported per 10 000 live births. We used Poisson distribution to calculate 95% CI of CHDs and its subtypes prevalence. Prevalence rate was used to describe the type-specific difference between urban and rural areas. The significance of categorical variables was assessed by a χ^2^ test or Fisher exact test. We used Cochran-Armitage trend test for analysis of trend. Odd ratio (OR) was presented. All ORs were calculated relative to rural areas. A probability value of *P* < 0.05 was regarded as significant. Statistical analysis was conducted using SPSS for Windows version 13.0.

## Results

### Demographic characteristics

Between 2008 and 2012, a total of 1005052 deliveries were reported to the Birth Defects Monitoring Network of Guangdong Province, of which 5268 cases were diagnosed as CHD. Baseline demographic characteristics of infants with CHD and their mothers are presented in Table 1.

**Table 1:** Demographic characteristics of perinatals with CHD and their mothers in Guangdong, China, 2008-2012

**Characteristics**	
**Maternal age, y**	28.19 ± 4.92
**Education**	
Illiterate	29 (0.55%)
1 ~ 6 years	172 (3.26%)
7 ~ 9 years	1771 (33.62%)
10 ~ 12 years	1939 (36.81%)
13 ~ 16 years	1347 (25.57%)
Unknown	10 (0.19%)
**Gestational age (week)**	
<37	1841 (34.95%)
≥37	3427 (65.05%)
**Fetal sex**	
Male	2975 (56.47%)
Female	2649 (43.30%)
Unknown	76 (0.23%)
**Birth weight (g)**	2748.00 ± 795.15
**Fetal number**	
Singleton	4979 (94.51%)
Twin	303 (5.30%)
Multi-fetal	10 (0.19%)

Data are expressed as mean ± standard deviation or no. (%).

### Birth prevalence

The overall birth prevalence of CHD was 52.41 per 10 000 births (95% CI: 51.00 ~ 53.83) in provincial-wide, 66.08 per 10 000 births (95% CI: 63.77 ~ 68.39) in urban areas, and 40.23 per 10 000 births (95% CI: 38.52 ~ 41.93) in rural areas.

The birth prevalence of CHD in urban areas increased from 59.33 per 10 000 urban births in 2008 to 107.78 per 10 000 urban births in 2012. In rural areas, the birth prevalence increased from 27.24 per 10 000 births in 2008 to 69.40 per 10 000 births in 2012. The results were shown in Table 2.

**Table 2:** Time trends in the prevalence of CHD (per 10 000 births) by rural–urban residence in Guangdong province, China, 2008-2012

**Year**	**Urban**			**Rural**			**Urban–rural rate ratio**
	**Birth**	**Cases**	**Prevalence (95% CI)**	**Birth**	**Cases**	**Prevalence (95% CI)**	
2008	83433	495	59.33 (54.12 ~ 64.54)	92131	251	27.24 (23.88 ~ 30.61)	2.18
2009	85413	405	47.42 (42.81 ~ 52.02)	97558	236	24.19 (21.11 ~ 27.27)	1.96
2010	92701	351	37.86 (33.91 ~ 41.82)	100417	373	37.14 (33.38 ~ 40.91)	1.02
2011	97024	638	65.76 (60.67 ~ 70.84)	112746	386	34.23 (30.83 ~ 37.64)	1.92
2012	115238	1242	107.78 (101.82 ~ 113.74)	128391	891	69.40 (64.86 ~ 73.94)	1.55
2008-2012	473809	3131	66.08 (63.77 ~ 68.39)	531243	2137	40.23 (38.52 ~ 41.93)	1.64

Note: CI = Confidence interval.

Maternal age was divided into five different age groups: <20 years, 20–24 years, 25–29 years, 30–34 years and ≥35 years. The birth prevalence of CHD increased with maternal age both in urban areas (*P* < 0.01) and in rural areas (*P* < 0.01). The results were shown in Table 3.

**Table 3:** Maternal age-specific disparity of prevalence of CHD between rural and urban areas in Guangdong, 2008-2012

**Age**	**Urban**			**Rural**			**Provincial**		
	**Cases**	**Prevalence**^**a**^	**95% CI**	**Cases**	**Prevalence**^**b**^	**95% CI**	**Cases**	**Prevalence**^**c**^	**95% CI**
<20	33	47.37	31.24 ~ 63.49	49	32.27	23.24 ~ 41.29	82	37.02	29.02 ~ 45.02
20-	547	57.49	52.68 ~ 62.29	571	39.16	35.96 ~ 42.37	1118	46.40	43.69 ~ 49.11
25-	1365	63.19	59.85 ~ 66.53	845	37.59	35.06 ~ 40.12	2210	50.14	48.05 ~ 52.22
30-	811	72.99	67.99 ~ 78.00	447	43.59	39.56 ~ 47.62	1258	58.88	55.64 ~ 62.13
≥35	375	84.13	75.65 ~ 92.60	225	52.40	45.57 ~ 59.23	600	68.56	63.09 ~ 74.03

Note: Note: CI = Confidence interval.

Prevalence trend was tested by Cochran-Armitage trend test.

^a^Z = −5.99, *P* < 0.01; ^b^Z = −3.86, *P* < 0.01; ^c^Z = −8.70, *P* < 0.01.

## Discussion

CHD is the most common group of congenital anomalies and a major cause of death in infancy and childhood [15]. They are frequently required multiple hospitalizations and surgical procedures. Our study examined the epidemiologic features of CHD, using the data from the Guangdong Hospital-Based Birth Defects Monitoring System. We provide birth prevalence for CHD. In our study, all live births and still births born in monitoring hospitals were included and accessed within 7 days after delivery. So the prevalence of CHD in our study reflects the perinatal prevalence of CHD.

Our results showed that the prevalence rises in urban and rural populations during 2008–2012. There are might three reasons for that. First, the use of ultrasonic examination makes the rate of congenital diagnosis improved. In the lack of ultrasonic examination, the percentage of CHD was lower [16]. In our study, we also found that the use of ultrasonic examination rises during 2008–2012 (data not shown). Second, with the improvement of congenital diagnosis, more and more CHD cases can be found in the perinatal period, resulting in an increasing prevalence. Parents accepted cardiac echocardiography as the most important neonatal screening method, and they were willing to screen their babies with cardiac echocardiography to determine whether CHD were present. Third, environmental and genetic factors can influence the prevalence of CHD.

The difference of maternal age-specific overall prevalence was observed in our study, and the prevalence in urban and rural areas increased with maternal age. A similar finding has been reported by Miller et al. [17], who reported that the prevalence in infants born to mothers older than 35 years was much higher than in those born to younger mothers. Other studies have showed that the prevalence of CHD increases with increasing maternal age [14,18,19].

In the birth defect registry system, the urban–rural classification depends on the place where the mother lived during the pregnancy, which mainly reflects the combined exposure during the pregnancy [18,20]. In our study, we found a higher prevalence in urban areas compared with rural areas. The education, economic level, occupational exposure, lifestyle, and health care are different between people who live in urban and rural areas in China [21,22]. On the other hand, living in rural areas may be associated with lower access to health care and a shortage of healthcare providers. The prevalence of CHD subtypes were also found to vary by urban–rural classification.

The data of our study is reliable and representative. The study was based on the birth defect registry system of the province of Guangdong, and the system contains 58 hospitals covering districts, which represents the total situation of CHD in Guangdong province well. All data were reported on the network in time. Strict quality control of data collection was taken by county, municipal and provincial experts every year, thus ensuring reliable data. However, several limitations in the study should be addressed. First, according to the surveillance system, the monitoring period was 28 weeks’ gestation to 6 days after delivery, and babies with CHD detected more than 7 days after delivery would be missed. Second, the study did not cover cases of births given out of hospital, although the proportion was small. Third, our study was difficulty in distinguishing between an ASD and a patent foramen ovale in the perinatal period. And because of most of foramen ovale and ductus arteriosus after birth to 6 days is normally patent and will be closed naturally within several months. The exclusion of a patent foramen ovale and patent ductus arteriosus in perinatal infants eliminated perinatal conditions from consideration, thereby improving the prevalence estimates of CHD. Finally, it is possible that hospital-based samples might introduce referral bias. However, our study used the data from the Guangdong Hospital-Based Birth Defects Monitoring System, which is covering 58 hospitals across the province, and our results provide value insights to our understanding of the main epidemiologic characteristics of CHD prevalence in Guangdong province.

The findings from our study have some practical implications. High prevalence of CHD indicates that CHD become one of the major public health concerns in Guangdong province, China. A high and increasing rate of CHD among perinatal infants increases the burden on families and health care providers, both in terms of parental anxiety, and of the direct costs of diagnostic examinations, follow-up, and possibly treatment [17].

## Conclusions

In conclusion, the birth prevalence of CHD increased from 2008 to 2012 both in urban and rural areas. The increasing trends of CHD prevalence suggest that maternal age and the improvement of diagnosis ability might play a critical role. More comprehensive surveillance among infants would improve prevalence rate for CHD and help assess prevention efforts, and knowing the prevalence and distribution of CHD can help pediatricians and public health professionals better assess health care needs and conduct causal research.

## Competing interests

The authors have declared that no competing interests exist.

## Authors’ contributions

QZ and BL designed the study and performed the analysis. SL and YM collected the data. ZL, TC and CJ assisted in the analysis and data interpretation. LW drafted the manuscript. JX and YW reviewed and refined the manuscript. All authors read and approved the final manuscript.

## Pre-publication history

The pre-publication history for this paper can be accessed here:

http://www.biomedcentral.com/1471-2458/14/152/prepub
